# Regional variation in the utilization of nursing home care in Germany

**DOI:** 10.1007/s10198-024-01732-9

**Published:** 2024-11-25

**Authors:** Annika Herr, Maximilian Lückemann, Amela Saric-Babin

**Affiliations:** 1 Institute of Health Economics (IHE), Leibniz Universität Hannover, CHERH Und CINCH, Königsworther Platz 1, 30167 Hannover, Germany; 2 CINCH, Universität Duisburg-Essen, Duisburg, Germany; 3 Düsseldorf Institute for Competition Economics (DICE), Heinrich-Heine-Universität, Düsseldorf, Germany; 4 Frankfurt, Germany

**Keywords:** Regional variation, Long-term care, Spatial panel data models, Nursing home, I11, I18, C23

## Abstract

Approximately 32 percent of individuals aged over 64 years old, with care needs, are residing in nursing homes in Germany. However, this percentage exhibits significant regional disparities, ranging from under 15 percent in certain counties to over 50 percent in others. The purpose of this study is to elucidate the underlying factors explaining this regional variation in nursing home utilization. We employed comprehensive administrative data encompassing the entire elderly care-dependent population and all nursing homes. Our analytical approach involves the use of linear regression models at the county level, accounting for an extensive array of control variables and fixed effects. Additionally, we analyzed regional dependencies by applying spatial lag models. In summary, our model successfully predicts up to 73 percent of the observed regional variation in nursing home utilization. Key factors include care needs, the presence of informal care support and the supply of professional care. Spatial dependencies can be detected but exhibit a minor influence on these variations controlling for care needs. Noteworthy, enabling factors, such as a region’s wealth or rurality, have a very limited impact in a country with a generous social insurance system that covers care for those with limited financial resources.

## Introduction

When it comes to choosing the best type of care, in many countries individuals with care needs have three options: (i) informal care by family members, or (combined with) (ii) formal care by home care providers in their homes, or (iii) nursing home care (NHC). In Germany, home care is prioritized over NHC as per the long-term care (LTC) system’s organizational principle, aiming to keep care recipients in their familiar environment for as long as possible (SGB XI §3). Despite the national regulation of the mandatory LTC insurance, there is significant regional variation in the utilization of nursing home care (NHC) in Germany. The unadjusted mean share varies from below 15 percent to more than 50 percent of all people over 65 years old and in need of care living in nursing homes (NHs) across the more than 400 German counties, with an average of 32 percent.

Understanding the disparities in NH utilization is crucial due to substantially higher public and private expenditures for NHC compared to home care or informal care. In 2021, the annual costs for NH were, on average, e21*,* 000 per person compared to e9*,* 400 for home care [1]. Furthermore, since demand for LTC is increasing, understanding the regional differences is essential for shaping future LTC policies.

Numerous papers have explored regional heterogeneity in healthcare utilization. One prominent example is Finkelstein et al. [2], who explore patient migration and find that a significant proportion of the variation in healthcare utilization can be attributed to demand factors such as preferences and health, besides the major impact of the place-specific supply factors that have been previously identified. Cutler et al. [3] argue that in the physician market, supply-side characteristics play a more substantial role compared to demand in explaining the regional heterogeneity in expenditures in the US. Godøy and Huitfeldt [4] highlight the influence of socioeconomic factors in explaining regional variation in healthcare utilization and mortality in Norway. Berger and Czypionka [5] show that demand-side factors explain most of the variations in magnetic resonance imaging across medical practices. Reich et al. [6] explore disparities of regional healthcare expenditures in Switzerland and present evidence for its correlation with supply-side densities and socioeconomic factors.

Regarding Germany, Augurzky et al. [7] examine the regional differences in the utilization of hospitals, Kopetsch and Schmitz [8] analyze the variation in the usage of ambulatory physician services, and Göppfahrt et al. [9] focus on total healthcare expenditures. Ozegowski and Sundmacher [10] analyze the discrepancy between regional needs and the utilization of outpatient care. They identify supply factors as the primary contributors to this gap. Some of the studies apply spatial autoregressive models and show that correlations are significant but small across counties. In contrast, Felder and Tauchmann [11] generate district-level efficiency scores in health production and highlight the importance of accounting for spatial dependence to analyze the strong effect of federal state-specific regulations on the district’s efficiency. All studies, as is common in this literature, present correlations between the outcomes and the regional explanatory factors rather than causal statements. One prominent exception is Salm and Wu¨bker [12] who utilize individual exogenous patient migration as an instrument to investigate regional differences in ambulatory care utilization. They identify demographics and patient characteristics as being most relevant, in contrast to institutional differences.

In terms of LTC provision, Pilny and Stroka [13] examine how the regional availability of NHs affects elderly care decisions using a discrete choice setting where individuals can choose between four different types of formal and informal care. Their study employs resident-level administrative data obtained from a large German health insurance. They find that the decision to choose NHC is significantly driven by the regional supply of NH beds. Mennicken et al. [14] focus on the differences in remuneration rates among NHs in North Rhine-Westphalia. They find that approximately 70 percent of the regional price differences can be explained, the largest part by regional negotiation styles between NHs and the different LTC insurance providers. Lastly, Duell et al. [15] examine the regional variation in the eligibility of publicly financed home care in the Netherlands, which is mainly driven by the place of residence rather than patient experiences.

Our study is the first to analyze spatial variations in NHC utilization. We contribute to the existing literature by integrating the healthcare services utilization model proposed by Andersen and Newman [16]. The model comprises three categories: individual determinants (needs, predisposing factors, and enabling factors), health services supply, and societal determinants. We adjust the model for the German LTC setting. We distinguish between individual care needs, the existence of informal care (predisposing), wealth-related factors (enabling), supply measures including prices, and societal determinants.

We use comprehensive data combining the German Care Statistic with regional socioeconomic and demographic data at the county level from 2007 to 2019 (biannually). The Care Statistic of the German Statistical Offices of the Länder comprises the entire German care-dependent population and all care facilities. Our methodological approach follows the small area variation studies by Cutler and Sheiner [17] on healthcare expenditures, successfully applied to other German healthcare markets by Augurzky et al. [7], Kopetsch and Schmitz [8], and Ozegowski and Sundmacher [10]. We estimate ordinary least squares (OLS) models with regional-fixed effects and wave-fixed effects to uncover the variation of our outcome variable. To account for spatial dependencies in utilization and regional shock spillovers inspired by Gupta et al. [18] and Ozegowski and Sundmacher [10], we additionally apply spatial autoregressive (lag) models.

Overall, our model achieves a good measure of fit explaining 73 percent of the variation in NH utilization across the more than 400 German counties, of which almost 64 percent can be attributed to care needs (explaining 47 percent of the variation). This is more than previous studies have found for other markets. Regional predisposing indicators, such as informal care opportunities measured by the female workforce and demographics, account for an additional 8–12 percentage points (depending on the order of inclusion). Regional enabling factors, including wealth and rurality, make a very small contribution, explaining an additional 1–2 percentage points of the variation. The supply of healthcare adds 5–10 percentage points when controlling for care needs, which is much less than in previous studies. Regional and wave-fixed effects finally explain 7 percentage points of the variation (added at last to the model). Furthermore, we identify a small yet significant presence of spatial dependencies that do not alter the main conclusions.

The findings have important policy implications since they demonstrate that besides the care needs, the degree of informal care support correlates highly with the demand for NHC. Thus, policymakers could either stimulate informal caregiving in some areas by reducing the double burden of work and caring or, in turn, increase the availability of NHC to reduce the need for informal care provision. It is also important to note that wealth and income cannot explain the variation even when not controlling for the supply of formal care that may be correlated.

Section "A model of health services utilization" discusses the model of health services utilization we apply to this setup, followed by Section "Data and descriptive statistics”, which presents the data and descriptive statistics. We introduce the estimation strategy in Section "Estimation strategy” and then present our results in Section "Results”. In Section "Discussion and conclusion”, we discuss our findings and conclude.

## A model of health services utilization

We examine the regional disparities in the utilization of NHC by employing a modified version of the Andersen-Newman model of healthcare utilization [16]. The model distinguishes between Individual determinants of utilization individual determinants, Health services supply supply of LTC services, and Societal determinants societal determinants of utilization. All parameters are aggregated at the county level. The individual characteristics from the care statistics are based on all elderly individuals (aged 65 or older) receiving any contribution from the LTC insurance system (across the three care types if not indicated differently). For an overview, all variables are grouped and defined briefly, including sources, in Table 5 in the appendix. 

### Individual determinants of utilization

As individual determinants of utilizing healthcare services are multi-faceted, we specify three groups of personal characteristics: care needs, predisposing factors, and enabling factors.

**Fig. 1:**
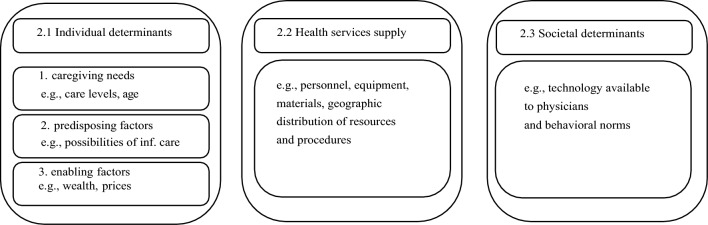
Determinants of the Andersen-Newman model of healthcare utilization Illustration of the three indicator groups derived from the Andersen-Newman model of healthcare utilization: (1) individual determinants, (2) health services supply, and (3) societal determinants

**Care needs** correspond to individual disabilities or health status. With regard to care needs, we include the average county-level care level it corresponds to. German regulations distinguish between three levels of LTC severity in our data.^1^ The need for caregiving arises if individuals require assistance with the daily living activities due to advanced age, health- or mental-related problems for a minimum of six months (SGB XI §14), which is assessed by external bodies of the mandatory LTC insurance, the so-called Medical Review Boards (Medizinischer Dienst der Krankenkassen [MDK] in German). They also set rules to negotiate prices between the providers and the statutory LTC insurances and communities and monitor quality on behalf of the health insurance providers (Fig. 1). They operate at the federal state level except for North Rhine-Westphalia (separated into North Rhine and Westphalia-Lippe), Hamburg (combined with Schleswig-Holstein), and Berlin (combined with Brandenburg).^2^ People who have been assigned a care level receive financial support from their LTC insurance (see respective allowances in Table 6 in the appendix). Level 1 indicates moderate needs while higher levels correspond to more severe health problems. Care recipients in care level 3 are disproportionately highly represented in NHs [19]. Age correlates with the emergence of frailties and therefore serves as a proxy for healthcare needs and patterns of medical care use [16]. We split the elderly care recipients (across all care types) into three groups: 65–74, 75–84 and 85 and over, where the lowest age group is the reference category. This follows the idea that older care recipients are more likely to use NHC than their younger peers [19, 20].

**Predisposing characteristics** capture the surrounding socio-demographic conditions. We employ several variables as proxies for the existence of informal care support: the shares of men and women in the active workforce, the regional demographics captured by the share of inhabitants in each age cohort, each with a different probability of serving as informal caregivers, and the average life expectancy at age 60. Labor force participation is defined as the share of men (or women) aged between 15 and 65 years in the active workforce. The effect of employment on the choice of the type of care is ambiguous. Employment reduces the capacity to provide informal care; yet flexible work arrangements (part-time, mini-jobs) can help reconcile work and care duties [21]. The propensity of daughters to provide informal care is generally higher than that of sons [22–25]. We expect that a higher share of women in the active workforce is associated with higher use of NHC, while the effect of men’s participation is not clear a priori. For example, women could partly substitute work with caregiving, while their spouses compensate for the earnings foregone by working more.

Around 48 percent of women in the active workforce in 2019 were part-time employed, while the corresponding share of men was only 11*.*2 percent [26]. We include the share of the population aged below 34 as reference category and add 35 to 49, 50 to 64, 65 to 74, 75 to 84, and 85 and older. The majority of caregiving relatives (children and their spouses) fall into the age span of 50–64. Augurzky et al. [7] suggest that most informal caregivers are between 55 and 69 years of age, which we approximate with the available data (50–65 years old). We also include life expectancy at age 60 and postulate that higher life expectancy is associated with better health in older ages, resulting in lower demand for NHC. However, having access to NHC might also increase life expectancy for the oldest care recipients.

**Enabling characteristics** predominantly cover the propensity of the affordability and accessibility of NHC. Here, we include GDP per capita, household income, average pension for seniors, rurality, the utilization of social aids for the elderly, and communal debts. Rurality serves as a proxy for travel times as distance influences facility choices [7, 27–29]. Rurality is measured as the share of the county’s population living in municipalities with fewer than 150 inhabitants per square kilometer. We postulate that NHC in Germany is utilized more frequently in urban than in rural areas. NHC is considered to be a normal good, meaning that demand is negatively affected by higher prices and positively by income and wealth. Therefore, we include various measures of income and impoverishment, such as the amount of social aid for the elderly and communal debts. Living in an NH is relatively expensive, with an average co-payment of e1*,* 620 per month (Table 1). In line with this, Bakx et al. [30] report that being in the bottom income quartile in Germany decreases the probability of using any formal LTC. Similarly, Augurzky et al. [4] and Eibich and Ziebarth [31] find that a higher income positively impacts the utilization of hospital and physician services.

**Table 1:** Descriptive statistics

	2007-2017			
	Mean	sd	p1	p99
Nursing home utilization				
Elderly care recipients in nursing homes [%]	31.89	7.10	17.93	49.97
Caregiving needs				
Elderly care recipients across all care types				
Share in care level 2	35.06	5.60	27.17	50.81
Share in care level 3	11.15	3.04	5.06	19.40
Share aged 75-84	43.69	3.89	33.74	52.55
Share aged 85+	43.69	3.89	33.74	52.55
Predisposing				
Labor force participation* [%]				
Female	76.69	4.90	67.00	85.25
Male	84.59	4.26	72.30	92.60
Inhabitants by age group [%]				
Age group 35–49	27.17	2.19	22.60	32.40
Age group 50–64	21.34	2.54	16.75	27.60
Age group 65–74	11.08	1.42	8.20	14.80
Age group 75–84	7.68	1.32	5.30	11.50
Age group $\ge$85	2.56	0.46	1.60	3.70
Avg. life expectancy at age 60[years]	83.41	0.678	81.97	84.98
Enabling				
GDP per capita [1000 EUR]	31.92	14.24	15.67	89.87
Use of social assistance for elderly [%]	19.28	12.67	3.80	61.80
Avg. pension [EUR]	833.83	84.02	659.50	1036.75
Monthly available household income [EUR]	1681.77	245.76	1228	2419
Share rurality**	0.29	0.29	0.00	1.00
Communal debts per capita [EUR]	1639.02	1365.52	0.00	6582.45
LTC supply				
Hospital beds [per 10,000 inh.]	6.44	3.84	0	19.29
Home care facilities [#]	34.49	41.26	7.00	180.00
Beds per residents (occupancy rate) in NH	0.89	0.06	0.74	0.99
Single-room share in NH [%]	61.12	11.06	36.02	85.18
Nurse vacancy ratio****	0.035	0.02	0.01	0.11
Personnel per resident, share	0.62	0.09	0.39	0.82
OOP*** care level 1 [EUR]	1436.82	316.40	719.29	2289.61
OOP*** care level 2 [EUR]	1572.76	334.09	809.01	2295.09
OOP*** care level 3 [EUR]	1788.19	368.65	1015.41	2535.70
Observations	2,221			

We report descriptive statistics for all German elderly care-dependent individuals (aged 65+) entitled to public and private long-term care insurance allowances aggregated at the county level for the years 2007, 2009, 2011, 2013, 2015, 2017

Sources: Research Data Centre (RDC) of the Federal Statistical Office and Statistical Offices of the Länder, Care Statistic, 2007 and 2017 (DOI: 10.21242/22411.2007.00.02.1.1.0–10.21242/22411.2017.00.02.1.1.0) [32]; INKAR database of the Federal Office for Regional Planning [26]; IAB administrative vacancy information from the Research Institute of the Federal Employment Agency [35]; Transparency report cards from the BKK comparison engine for stationary LTC https://pflegefinder.bkk-dachverband.de/. Own calculations

*Labor force participation is a share of males/females in the age group 15–65 in the active workforce

**Rurality is defined as a share of county’s population living in municipalities with less than 150 residents per km$^2$

***OOP refers to a price negotiated for each nursing home net of the allowance paid by the mandatory LTC insurance

****Vacancy ratio is the ratio of open positions to the total number of budgeted positions

***** Nursing care quality is an indicator based on the quality report cards of the Medical Review Boards (MRB), between zero (very poor quality) and one (excellent quality) (see A1 for details in the Appendix).

### Health services supply

Health services supply encompasses the resources and organization of healthcare delivery. This category includes personnel, equipment, materials, resources, and procedures employed once a person in need of care becomes part of the LTC system. To approximate the regional supply of healthcare services, we consider various provider characteristics. Firstly, we include the number of hospital beds per 10,000 inhabitants, which can potentially serve as a temporary alternative for NHC, especially following surgical interventions in seniors [32]. Secondly, we postulate that the availability of home care may also contribute to delaying the utilization of NHC. To assess the supply of NHC, we include the average single-room share, the personnel vacancy ratio, the occupation ratio, and the personnel-to-resident ratio. We include NH prices by care level 1 to 3 as the log of the out-of-pocket payment, i.e., the share of the price that is not covered by the LTC insurance (either financed privately out-of-pocket or, if not feasible, covered by the social insurance system). Lastly, we control for the NH’s quality by including a quality indicator (see the appendix for the definition).

### Societal determinants

Finally, societal determinants of NH choice include the technology available to physicians and behavioral norms. Societal determinants of utilization are generally not observable [31]. We hypothesize that the regional fixed effects capture not only the geographic differences in technology but also differences in behavioral norms.

## Data and descriptive statistics

We exploit three data sources.

First, we use the German care statistics from the Federal Statistical Offices and the Statistical Offices of the Länder at the Research Data Center (RDC) of Hannover (“Care Statistics”). The bi-annual statistic spans over six waves from 2007 to 2017, reflecting the German care situation on December 15 every two years. The data comprise all care recipients entitled to public or private LTC insurance allowances in Germany. This includes both informal and professional care recipients, where the latter are differentiated between NHC and home care. The data also cover all NHC and home care providers, e.g., the number of available places, the personnel, the type of caregiving services offered, and the fees to be paid to the care facility for caregiving, accommodation, and meals. To focus on regional variation rather than individual determinants of NH entry, we aggregate the data at the county level.

Second, we supplement the care statistic with indicators of regional and urban development, such as socio-economics, demographics, and health care supply, at the county level provided by the Federal Office for Building and Regional Planning (INKAR) [7] (in German: Indikatoren und Karten zur Raum- und Stadtentwicklung [INKAR], https://www.inkar.de/.) Third, we add labor market information from the German Institute for Employment Research, more specifically, the number of vacancies for nurses in geriatric care per county and year [41].

In our final sample, we excluded all people under 65 years of age in need of care and also all care facilities that provide non-elderly care (e.g., care for kids or psychiatric care) or facilities that provide only short-term care or only day or night care. In compliance with the data security rules enforced by the research data center, we aggregated neighboring counties with fewer than three NHs per ownership type and county, which gives us 374 (2007) and 367 (2017) regions covering all 400 counties across the 16 federal states (2221 observations over six waves). The dependent variable is defined as the county’s share of elderly care recipients aged 65 or older living in NHs as opposed to living at home (receiving only informal care or home care support).

**Fig. 2:**
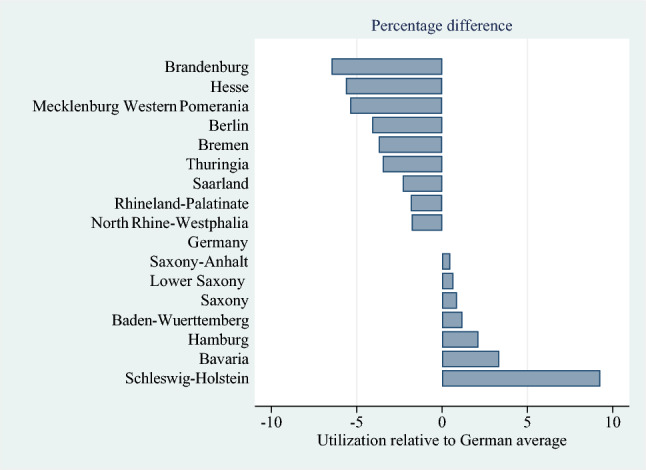
Nursing home (NH) utilization across federal states compared to the German average. *Data source*: Statistical Offices of the Länder, Care Statistic, 2007 to 2017 (DOI: 10.21242/22411.2007.00.02.1.1.0–10.21242/22411.2017.00.02.1.1.0). *Note:* This figure shows average federal-state deviation from our main dependent variable mean over time from 2007 to 2017. The average share of care recipients moving into NHs is appr. 31%. Deviations are provided in percentage points

Figure 2 demonstrates regional disparities in the utilization of NHC across federal states. NHC is most frequently used in Schleswig-Holstein, Bavaria, and Hamburg. In Schleswig-Holstein, the proportion of care recipients in NHs is consistently 9–10 percentage points higher than the national average. Conversely, Brandenburg, Hesse, and Mecklenburg Western Pomerania fall 5–6 percentage points below the average.

The appendix provides maps that visualize the regional variation in LTC utilization across care types and its changes over time. From these maps, we can discern certain patterns. Figure 4 illustrates the variation in NHC across counties in 2007 and 2017. NHC appears to be more prevalent in the northern and southern regions of Germany. For the two other care types, there is a higher utilization of home care in the northeast (Figure 5) and greater reliance on informal care in the southwest (Figure 6).

**Fig. 3:**
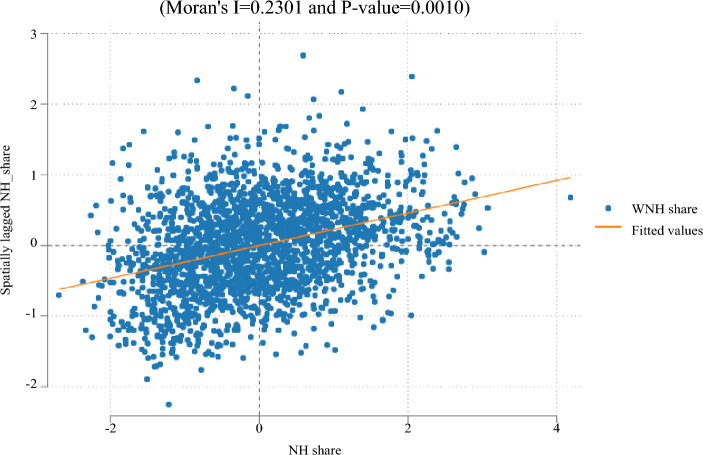
Moran scatter plot. *Data source*: RDC of the Federal Statistical Office and Statistical Offices of the Länder, Care Statistic, 2007 and 2017 (DOI: 10.21242/22411.2007.00.02.1.1.0–10.21242/22411.2017.00.02.1.1.0), own calculations. *Notes*: The Moran scatter plot displays how the selected attribute’s values at each location relate to the average value of the same attribute at neighboring locations. The upper-right (lower-left) quadrant represents cases where both the attribute value and the local average value exceed (lie below) the overall average value, indicating positive spatial autocorrelations. The other two quadrants indicate negative spatial autocorrelations. The dominant groups within these quadrants determine the overall tendency toward positive, negative, or no spatial autocorrelations (see, e.g., lecture notes from Penn State, Project 4: Calculating Global Moran’s I and the Moran Scatter plot, https://www.e-education.psu.edu/geog586/node/672, last accessed in July 2024)

Table 1 summarizes all variables included in our model. For detailed variable definitions compare Table 5 in the appendix. The table shows that there is strong regional variation across all variables when looking at the lowest and highest percentile.

## Estimation strategy

### Multivariate linear regressions to explain variation in NHC

Our estimation approach follows Cutler and Sheiner [17], Kopetsch and Schmitz [8], and Berger and Czypionka [5] and allows us to net out the variation that is due to systematic differences between the counties. We gradually add groups of explanatory variables to the regression and infer their explanatory power from the changes in goodness-of-fit measures. Our preferred order of inclusion corresponds to the models presented in Section "A model of health services utilization".

The simple linear regression equation is given as

1 $${y}_{ct}= {X}_{ct}{\prime}\beta + {R}_{r} + {\tau }_{t}+ {\mu }_{ct}$$

where *y*_*ct*_ is the share of care recipients in NHC in county *c* and wave *t*. *X* varies across models depending on the control variables included in groups. We start with care needs and subsequently include predisposing, enabling, and supply factors. Finally, in the full model, we add wave-fixed effects *τ*_*t*_ and regional fixed effects *R* to account for unobservable factors at the relevant administration level *r*, which are the Medical Review Boards (MRB or MDK in German) since they set the rules to negotiate prices and monitor quality on behalf of the health insurance providers as discussed above. *µ*_*ct*_ indicates the IID disturbances term. The standard errors are clustered by region and time (MRB times year).

Since the explanatory power of each variable block varies based on the sequence of inclusion, we also specify alternative sequences for robustness analyses (compare section “Alternative Ordering”).

### Spatial auto-correlation across counties

As German counties cluster in bigger regions, e.g., federal states, there may be correlations in healthcare use across counties. That is why, in a second step, we account for confounding spatial dependencies by employing spatial regression models and test whether the coefficients of the explanatory variables change compared to the non-spatial linear regressions.

In spatial interaction-based models, collective behaviors and aggregate patterns are assumed to emerge from the interaction of agents across social, economic, and geographic dimensions [35, 36]. Interaction can be (a) endogenous, where the group causally influences individual behavior, (b) exogenous, where individual behavior varies with exogenous characteristics of the group, or (c) correlated, where similar behavior is due to similar individual characteristics and institutional environments [37]. The underlying idea is that actions chosen by one individual influence the constraints, expectations, and preferences in her reference group [38].

Endogenous interactions in NHC may arise from cultural factors. For example, the high use of NHC may increase its broader societal acceptance. In our context, exogenous interaction can be attributed to a wider effect of local developments. For example, the closure of a large NH in one county is likely to boost the demand for NHC in neighboring counties. Negative economic shocks will increase unemployment, which could increase the degree of informal care support. Correlated interactions result from factors that cannot be observed in the data. Examples include a high prevalence of conditions that often precede moving to an NH, such as mental diseases and strokes, or a good quality of care in a particular region.

We follow Kopetsch and Schmitz [8] and Gupta et al. [18], who analyze spatial dependencies in other healthcare markets, and assume that the use of NHC follows a spatial autoregressive process. Equations (2) and (3) formally describe the estimation procedure of the spatial autoregressive combined model (SAC), including a spatially lagged outcome and error term:

2 $${y}_{ct} = \lambda W{y}_{jt} + {X}_{ct}{\prime}\beta + {R}_{r} + {\tau }_{t} +{\mu }_{ct}$$

3 $${u}_{ct} = \rho {Wu}_{jt} +{\epsilon }_{ct},$$

where *W* originates from the spatial contiguity weight matrix, and ${\mu }_{ct}$ and ${\epsilon }_{ct}$ are vectors of spatially correlated residuals and IID disturbances, whereby ${\epsilon }_{ct}$ ∼ *N* (0*, σ*^2^I). We denote $W{y}_{jt}$ and ${Wu}_{jt}$ as spatial lags of the dependent variables and regression residuals, respectively. The subscript *j* captures the neighboring counties. The spatial contiguity weight matrix *W* parameterizes the interaction between the counties, i.e., it captures the influence of the use of NHC in county *j* on county *i*. We adopt a geographical contiguity criterion and assign *W* = 1 if counties share a common border or if the distance between their centroids is less than 60 km, and 0 otherwise [39].^3^ Coefficient *λ* captures the relationship between the use of NHC in nearby counties conditional on explanatory variables. Coefficient *ρ* captures the spatial correlation of demand shocks and unobservable factors in nearby counties. The spatial contiguity weight matrix is generated using the spmat command in Stata 17 [40]. Since the majority of LTC regulations are delegated to the federal state or local MRB, we include fixed effects at the MRB level. In addition, we capture potential changes in demand or supply over time by including wave-fixed effects. Standard errors are robust to heteroscedasticity and again clustered at the MRB-wave level.

In addition to the SAC, we estimate two more simplified spatial autoregressive models: the spatial lag model (SLM) and the spatial error model (SER) [41]. The SLM includes only a lagged outcome variable (see Eq. (2)), while the SEM has only a lagged error term (a combination of Eqs. (1) and (3)). These simplifications rule out the respective other type of spatial dependency.

## Results

### Explaining variation in NHC utilization

Table 2 presents the linear regression results. We gradually include blocks of explanatory variables following the sequence described in Section "A model of health services utilization". The explanatory power of each block is measured by the change in the adjusted r-squared (R^2^), which expresses the proportion of explained-to-unexplained variation in the dependent variable [42]. We can reject the multicollinearity of the different control variables using variance inflation factor (VIF) tests after the linear regressions (results upon request).

**Table 2:** Regression results on variation in NHC, 2007–2017

	Model 1	Model 2	Model 3	Model 4	Model 5
Elderly care recipients in level 2 [%]	– 0.0092	– 0.0307	– 0.0504	– 0.0995	0.0003
	(0.0590)	(0.0477)	(0.0511)	(0.0658)	(0.0687)
Elderly care recipients in level 3 [%]	0.5560***	0.4139***	0.3959***	0.1967***	0.2422***
	(0.0969)	(0.0513)	(0.0802)	(0.0729)	(0.0555)
Elderly care recipients aged 75 – 84 [%]	– 1.627***	– 0.7844***	– 0.8873***	– 0.7100***	– 0.4878***
	(0.1480)	(0.1816)	(0.1815)	(0.1447)	(0.1013)
Elderly care recipients aged 85+ [%]	– 0.211*	0.8343***	0.6711***	0.7135***	0.7842***
	(0.1201)	(0.1279)	(0.1297)	(0.1101)	(0.0926)
Predisposing					
Female labor force participation, share		0.2433***	0.2585***	0.1116*	0.2746***
		(0.0580)	(0.0593)	(0.0582)	(0.0591)
Male labor force participation, share		– 0.1135***	– 0.1671***	– 0.0949***	– 0.2011***
		(0.0414)	(0.0384)	(0.0336)	(0.0313)
Inhabitants 35–49, share		0.2404	0.1567	0.4522***	0.2393
		(0.1736)	(0.1770)	(0.1401)	(0.1554)
Inhabitants aged 50–64, share		– 0.9781***	– 0.9556***	– 0.8012***	– 0.3347***
		(0.1210)	(0.1260)	(0.1376)	(0.1032)
Inhabitants 65–74, share		0.5286***	0.3698**	0.2794	– 0.6543***
		(0.1835)	(0.1724)	(0.1994)	(0.1846)
Inhabitants 75–84, share		1.919***	1.626***	1.395***	1.544***
		(0.3374)	(0.4323)	(0.3940)	(0.2699)
Inhabitants $\ge 85$, share		– 4.083***	– 3.357***	– 3.256***	– 2.204***
		(0.8868)	(1.0100)	(0.8692)	(0.6460)
Avg. life expectancy at 60, years		– 0.0386***	– 0.0430***	– 0.0369***	– .0325***
		(0.0041)	(0.0041)	(0.0035)	(.003)
Enabling					
Log(GDP per capita [1,000 EUR])			0.0143**	– 0.0045	0.0011
			(0.0061)	(0.0057)	(0.0052)
Social aids for elderly [%]			– 0.0740***	– 0.0185	– 0.0059
			(0.0225)	(0.0239)	(0.0157)
Log (Avg. pension)			0.0166	0.0465	0.1589***
			(0.0278)	(0.0282)	(0.0268)
Share rurality**			– 0.0165*	– 0.0237***	– 0.0215***
			(0.0083)	(0.0072)	(0.0061)
Log (Available household income)			0.0229	0.0429**	0.0189
			(0.0177)	(0.0200)	(0.0174)
Log (Communal debts p.c. [EUR])			0.0007	0.0010	0.0002
			(0.0007)	(0.0010)	(0.0006)
Supply					
Hospital beds [per 10,000 inh.]				0.0022***	0.0022***
				(0.0004)	(0.0003)
Single-room share in NH [%]				– 0.0388***	– 0.0328**
				(0.0138)	(0.0136)
Home care facilities				– 0.0002***	– 0.0002***
				(0.00005)	(0.00005)
Occupancy rate in NH				– 0.1002***	– .1556***
				(0.0317)	(0.0280)
Nurse vacancy ratio****				– 0.4489***	– 0.3009***
				(0.0663)	(0.0590)
Personnel per resident ratio				– 0.0848***	– 0.1263***
				(0.0291)	(0.0188)
Log (Average OOP*** [EUR], level 1)				0.1002	0.0961*
				(0.0715)	(0.0489)
Log (Average OOP*** [EUR], level 2)				– 0.0347	– 0.1306
				(0.0846)	(0.0799)
Log (Average OOP*** [EUR], level 3)				– 0.1045***	– 0.0003
				(0.0333)	(0.0610)
Nursing care quality*****				0.0332**	0.0176*
				(0.0138)	(0.0092)
Constant	1.005***	3.403***	3.647***	3.228***	2.218***
	(.1115)	(0.3544)	(0.3667)	(0.3564)	(0.2441)
Observations	2221	2221	2221	2221	2221
MRB FE, Time FE	No	No	No	No	Yes
F-statistic	150.02	199.66	178.75	197.72	407.04
adj. R2	0.469	0.590	0.598	0.655	0.729

$$^{*}p<0.10$$, $$^{**}p<0.05$$, $$^{***}p<0.01$$; standard errors are clustered at the MRB $$\cdot$$ year level. Specifications: (1) caregiving needs; (2) + predisposing; (3) + enabling (without supply); (4) + enabling (with supply); (5) + federal state and year fixed effects. Sources: Research Data Centre (RDC) of the Federal Statistical Office and Statistical Offices of the Länder, Care Statistics, survey years 2007-2017 (DOI: 10.21242/22411.2007.00.02.1.1.0–10.21242/22411.2017.00.02.1.1.0); INKAR database of the Federal Office for Building and Regional Planning (BBR). Own calculations

Overall, including all controls and regional fixed effects (Model 5), we can explain 73 percent of the variation in the use of NHC, leaving 27 percent unexplained. Looking at the factors, we find that the proxies for care needs explain around 47 percent of the total variation (Table 2: Model 1), which corresponds to two thirds of the explained variation. Including predisposing variables raises the R^2^ to 59 percent (Model 2). Enabling factors add relatively little to the explanatory power of our model, increasing the R^2^ to 60 percent (Model 3). The supply factors add 6 percentage points, with an R^2^ of 66 percent (Model 4). Finally, county and wave-fixed effects increase the adjusted R^2^ to 73 percent (Model 5). The fixed effects capture, e.g., unobserved political and regulatory differences or differences in culture and individual preferences that do not change over time.

### Accounting for spatial autocorrelation in the preferred model

Model (5) explains most of the variation in NH use and it turns out that the goodness of fit of the five linear regression models changes similarly when controlling for autocorrelation such that we can only focus on Model (5) in the following [43]. Now, we test whether there is spatial autocorrelation in the utilization or errors of our regression model and, if yes, control for it.

To start descriptively, Figure 3 clearly illustrates the existence and the direction of spatial autocorrelations. Furthermore, the Moran-I coefficient of 0*.*23 rejects the null hypothesis of zero spatial autocorrelation across counties.^4^ Figure 8 shows the regional spatial correlations in a map where the red fields indicate negative and the blue fields indicate positive spatial dependencies by below or above-average utilization.

Next, we sequentially account for spatial dependencies as described in section “Spatial auto-correlation across counties” and estimate three different models (SLM, SEM, and SAC). Table 3 shows that spatial autocorrelation plays a small but significant role (see *λ* and *ρ*). Since both the spatial dependencies in utilization (SLM) and spatial correlation of shocks (SER) are significantly different from zero, we consider the SAC that combines both to be our preferred model. The full results can be found in Table 7 in the appendix.

**Table 3:** Spatial regression results based on OLS model (5), 2007–2017, shortened results

	SLM	SEM	SAC
Constant	2.3142***	2.1161***	2.1749***
	(0.201)	(0.209)	(0.207)
lambda ($\lambda$)	0.0060***		0.0065***
	(0.001)		(0.002)
rho ($\rho$)		0.0823***	0.0770***
		(0.006)	(0.006)
All controls (model 5)	Yes	Yes	Yes
MRB FE, Time FE	Yes	Yes	Yes
Observations	2221	2221	2221

$$^{*}p<0.10$$, $$^{**}p<0.05$$, $$^{***}p<0.01$$

Specifications: (1) Spatial Lag Model (SLM), Equation 2, (2) Spatial Error Model (SEM) Equation 3, (3) Spatial Autoregressive Combined Model (SAC), Equations 2 and 3.

Source: Research Data Centre (RDC) of the Federal Statistical Office and Statistical Offices of the Länder, Care Statistics, survey years 2007-2017 (DOI: 10.21242/22411.2007.00.02.1.1.0–10.21242/22411.2017.00.02.1.1.0); INKAR database of the Federal Office for Building and Regional Planning (BBR), own calculations. The full results can be found in Table 7 in the appendix

### Interpretation of interrelation between healthcare utilization factors and the NHC use in Germany

Since the OLS results of the preferred Model 5 in Table 2 are very similar to the SAC results in Table 7 in the appendix, we interpret both together.^5^

In both approaches, three of the four measures for care needs are correlated with NH utilization across all models.^6^ This is in line with several studies showing the care needs largely driving the decision to enter an NH [7]. Interpretation of the single coefficients is difficult, though, since they are correlated with each other (no exogenous variation). For example, the older care recipients are more likely to be classified as care level 3. Given the same care level, 75- to 84-year-old care recipients use NHC less often than their younger peers (65−74) (when not controlling for demographics, Model 1, also the share of oldest old above 84 is negatively correlated to NHC use). This may be due to younger care recipients at the same care level being more likely to suffer from physical impairments as opposed to, say, dementia, which makes them more likely to choose NHC at a younger age.

Second, predisposing characteristics are also very important. Controlling for all other factors, NHC is used intensively in counties with higher female labor participation. Women, especially in more traditional family roles, are more likely to care for their spouse or parents. A higher share of people aged 50–64 and 65–74 (compared to below 35) is related to a less intense use of NHC, which is in line with our hypothesis that this generation is very likely to care for their parents, while a higher share of people aged over 74 increases NHC use.

Third, only two of the enabling indicators correlate significantly with NHC use and the explanatory power of this group is very low. In our most comprehensive Model 5, as expected, pensions are positively correlated, while rurality is negatively correlated with NHC use. Without controlling for unobservable regional fixed effects, we find a weak association between higher household income and higher NHC use but we like to emphasize that GDP and communal debts are not significantly associated with NHC use, everything else equal. We conclude that NHC use is not directly linked to the county’s wealth.

Fourth, other factors held constant, the use of NHC is significantly higher in regions with a higher density of hospital beds. The number of home care providers is negatively correlated, however, it is negligible in magnitude. Other supply capacity measures, such as occupancy rates, single room shares, and nurse-vacancy ratios, reveal a substantial negative correlation with NHC use. In particular, the estimated coefficient for the nurse-vacancy ratio, a measure of nursing personnel shortage, is considerably large. This is connected to our quality measures as a higher personnel-to-resident ratio is associated with lower NHC use while nursing care quality is positively related to NHC use. Prices, however, do not play a large role.

Considering that we measure correlations, we cannot interpret the coefficients in a causal way. For example, since home care is usually combined with informal care support by relatives, its explanatory power might partly be captured by the predisposing variables.

### Alternative ordering

The explanatory power of individual variable blocks may change with the respective order of inclusion. Although we argue why we believe that the order is best as shown in our preferred model, we tried multiple alternative sequences (compare Table 4). While the respective explanatory power of each block varies, our main findings are confirmed. Always keeping the care needs as a basis, the additional explanatory power of predisposing variables lies between 8 and 12 percentage points. Enabling variables explain additionally 1–2 percentage points of the variation, while supply variables capture an additional 5–10 percentage points. Thus, on average, predisposing explains the highest proportion of the variation (additional to care needs), followed by supply measures. Regional enabling characteristics add very little.

**Table 4:** Explaining the variation in utilization of NHC, baseline vs. alternative ordering

		$R^2$	$\Delta$	$\lambda$	$\rho$
Baseline* specification					
Need		0.469	–	– 0.0097***	0.116***
				(0.0021)	(0.0020)
+ Predisposing		0.590	0.12	0.0009	0.108***
				(0.0020)	(0.0046)
+ Enabling		0.598	0.01	0.0035*	0.110***
				(0.0021)	(0.0045)
+ Elderly care supply		0.655	0.06	0.0056***	0.108***
				(0.0020)	(0.0046)
Alternative ordering 1					
+ Enabling		0.490	0.02	– 0.0020	0.116***
				(0.0022)	(0.0040)
+ Elderly care supply		0.578	0.09	0.0041**	0.114***
				(0.0021)	(0.0047)
+ Predisposing		0.655	0.08	baseline*	baseline*
Alternative ordering 2					
+ Elderly care supply		0.566	0.10	0.0010**	0.113***
				(0.0021)	(0.0049)
+ Enabling		0.578	0.01	0.0041**	0.114***
				(0.0021)	(0.0047)
+ Predisposing		0.655	0.08	baseline*	baseline*
Alternative ordering 3					
+ Enabling		0.490	0.02	– 0.0012	0.116***
				(0.0022)	(0.0040)
+ Predisposing		0.598	0.11	0.0035*	0.110***
				(0.0021)	(0.0045)
+ Elderly care supply		0.655	0.06	baseline*	baseline*
Alternative ordering 4					
+ Predisposing		0.590	.12	0.0009	0.108***
				(0.0020)	(0.0046)
+ Elderly care supply		0.644	.05	0.0043**	0.107***
				(0.0020)	(0.0047)
+ Enabling		0.655	.01	baseline*	baseline*
Alternative ordering 5					
+ Elderly care supply		0.566	.10	0.0043**	0.107***
				(0.0020)	(0.0047)
+ Predisposing		0.644	.08	0.0043**	0.107***
				(0.0020)	(0.0047)
+ Enabling		0.655	.01	baseline*	baseline*

$$^{*}p<0.10$$, $$^{**}p<0.05$$, $$^{***}p<0.01$$; Standard errors in parenthesis. *see *Elderly care supply* in baseline

Changes in adjusted $R^2$ when including blocks of explanatory variables in different orders. $\lambda$ and $\rho$ capture the spatial correlation as defined in the Spatial Autoregressive Combined Model (SAC), Equations 2 and 3. *Data sources*: Research Data Centre (RDC) of the Federal Statistical Office and Statistical Offices of the Länder, Care Statistics, survey years 2007–2017 (DOI: 10.21242/22411.2007.00.02.1.1.0–10.21242/22411.2017.00.02.1.1.0); INKAR database of the Federal Office for Building and Regional Planning (BBR), own calculations

**Fig. 7:**
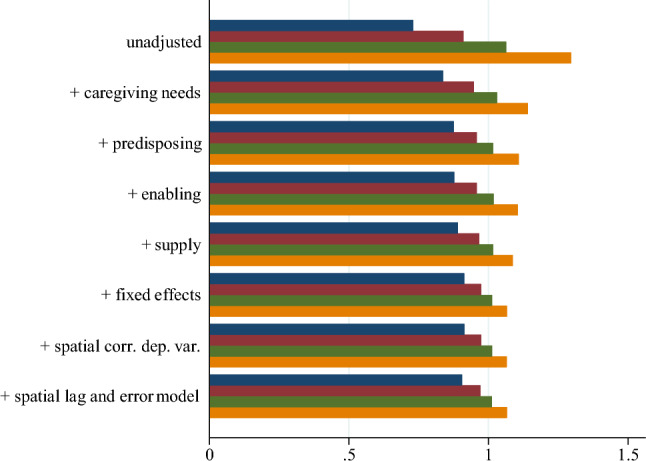
Observed-to-predicted ratio of NHC recipients by quartile of observed utilization. *Note:* We classify the counties into quartiles based on the unadjusted shares of care recipients in NH. blue: lowest 25%, red: 25–49%, green: 50–75%, yellow: highest quartile. The specifications used are as follows: 1. Unadjusted; 2. Adjusted for need; 3. Additionally adjusted for predisposing factors; 4. + enabling factors; 5. + NH supply; 6. + MRB and wave fixed effects; 7 + 8 + spatial correlations. *Data source*: RDC of the Federal Statistical Office and Statistical Offices of the Länder, Care Statistic, 2007 and 2017 (DOI: https://doi.org/10.21242/22411.2007.00.02.1.1.0–https://doi.org/10.21242/22411.2017.00.02.1.1.0). Own calculations

**Fig. 8:**
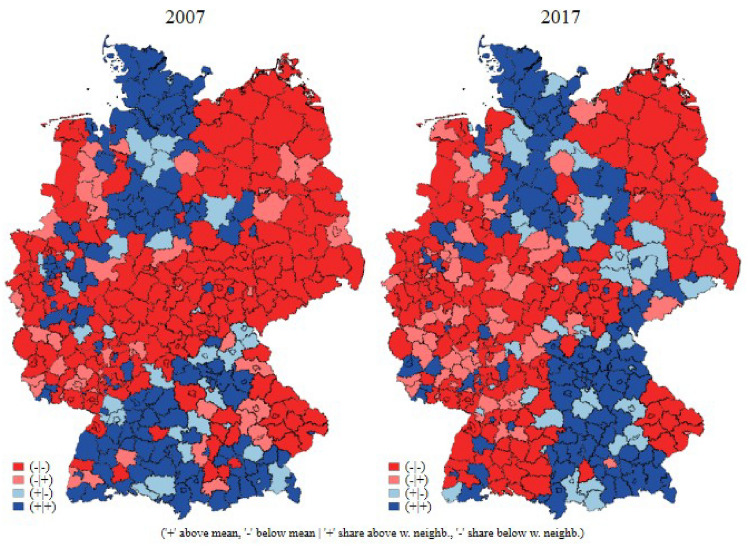
Shares of care recipients in NHC and spatial dependence. Average shares of care recipients in NH for 2007 and 2017 across counties above or below the national average and the sign of its spatial dependence. ( – , +) utilization below mean, positive spatial dependence; ( – , – ) utilization below mean, negative spatial dependence; (+ , – ) utilization above mean, negative spatial dependence; (+ , +) utilization above mean, positive spatial dependence. *Data source*: RDC of the Federal Statistical Office and Statistical Offices of the Länder, Care Statistic, 2007 and 2017 (DOI: https://doi.org/10.21242/22411.2007.00.02.1.1.0–https://doi.org/10.21242/22411.2017.00.02.1.1.0). Own calculations

To evaluate the model’s fit, Fig. 7 presents the ratio of the observed-to-predicted shares of care-dependent elderly in NHs by quartile of the observed share of NHC utilization [19]. The unadjusted model can predict neither very low nor very high utilization. The more indicators are added, the closer the predictions get to the observed values (getting closer to one from below and above).

## Discussion and conclusion

We investigate the factors contributing to regional variations in the utilization of nursing home care (NHC) for the elderly (65 years old and above) in Germany. Despite Germany’s comprehensive healthcare system, characterized by extensive coverage and mandatory long-term care (LTC) insurance, significant disparities in NHC usage exist across counties.

Applying the Andersen-Newman model of health services utilization [16], we categorize relevant factors into five groups: care needs (the primary determinants), predisposing factors, enabling factors, LTC supply, and unobserved differences at the regional administrative level. We are able to explain 73 percent of the regional variation in NHC utilization across the 400 German counties and 16 federal states.

Our initial linear regression analysis reveals that care needs and predisposing factors, such as the female workforce, life expectancy, and the proportion of the population in caring age, account for 59/73=80 percent of the explained variation in NHC use. Surprisingly, the supply of NH places plays a less crucial role than anticipated (additional 5–10 percentage points of the variation). Income, wealth, and rurality only contribute minimally.

Overall, our model demonstrates a satisfactory fit with an adjusted R^2^ of 73 percent, comparable to previous studies on regional variation in healthcare utilization [7–9]. Unexplained variation may stem from cultural differences, individual health characteristics, diverse working arrangements, family structures, or evolving population dynamics within counties.

In a second step, we explore spatial dependencies across counties to account for regional spillover effects. Although the hypothesis of no spatial autocorrelation is rejected, estimates from models with and without spatial dimensions remain mostly similar. The small spatial correlation coefficients lead us to conclude that spatial dependencies play a minor role in the LTC market when controlling for various determinants.

We utilize the German care statistics, covering the entire care-dependent population and all providers, mitigating issues associated with selected samples. Our proxies for care needs capture only a daily amount of required care without specifying impairments. Future research may delve into more detailed health characteristics, particularly distinguishing between physical and cognitive impairments.

Our results carry significant policy implications, emphasizing the critical role of informal caregiving. With the expanding care-dependent population, supporting and compensating informal caregivers becomes imperative. Implementing mixed solutions, such as assisted living, and redefining the role of NHs as a last resort could meet individual preferences and lead to a better allocation of resources.

Lastly, the negative relationship between capacity measures and NHC use suggests subsidizing NH expansion in low-supply areas. Strengthening rural areas, where a shortage of young trained nurses is prominent, is a proposed solution. Enabling factors like GDP, income, and rurality have minimal impact in Germany, making NHC use not income-dependent.

## Figures and Tables

**Fig. 4:**
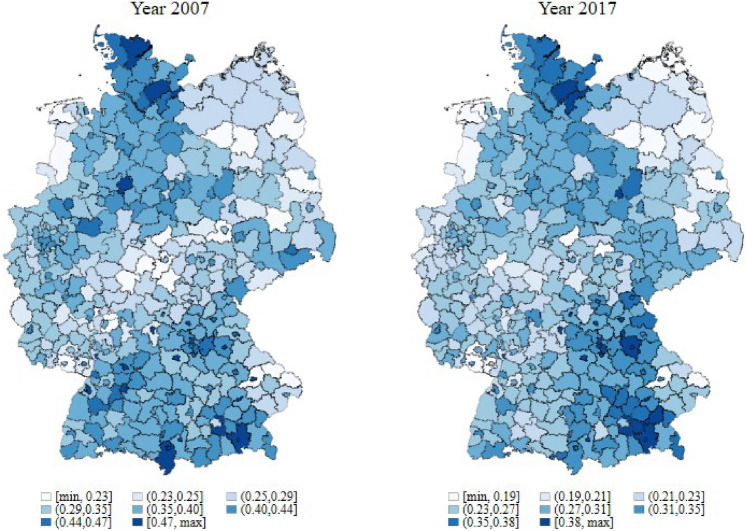
Share of elderly in nursing home care (NHC) to all elderly care recipients over 65 across German counties in 2007 and 2017. *Data source*: RDC of the Federal Statistical Office and Statistical Offices of the Länder, Care Statistic, 2007 and 2017 (DOI: https://doi.org/10.21242/22411.2007.00.02.1.1.0–https://doi.org/10.21242/22411.2017.00.02.1.1.0), own calculations. *Note:* Regional variation of the share of care-dependent elderly in NHC to all care recipients over 65 in Germany in 2007 (left) and 2017 (right) for different percentiles (p10, p25, p50, p75, p90). For data security reasons, we cannot show the maximum or minimum

**Fig. 5:**
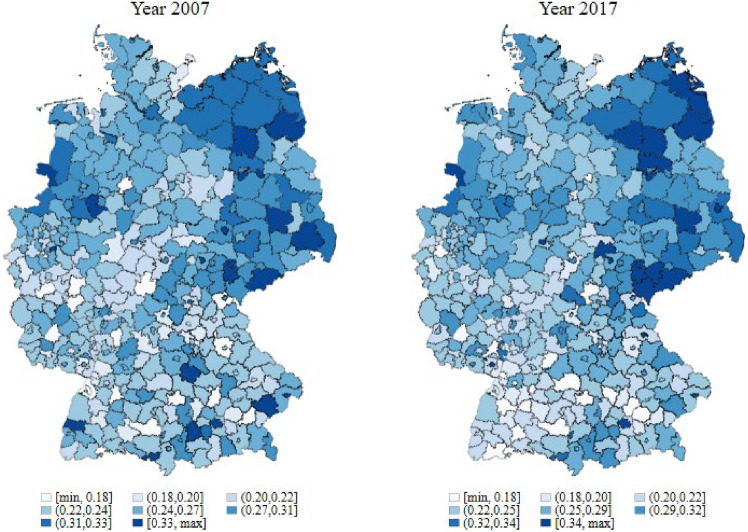
Share of elderly in home care to all care recipients over 65 across German counties in 2007 and 2017. *Data source*: RDC of the Federal Statistical Office and Statistical Offices of the Länder, Care Statistic, 2007 and 2017 (DOI: https://doi.org/10.21242/22411.2007.00.02.1.1.0–https://doi.org/10.21242/22411.2017.00.02.1.1.0), own calculations. *Note:* Regional variation of the share of people in home care to all care recipients in Germany in 2007 (left) and 2017 (right) for different percentiles (p10, p25, p50, p75, p90). For data security reasons, we cannot show the maximum or minimum

**Fig. 6:**
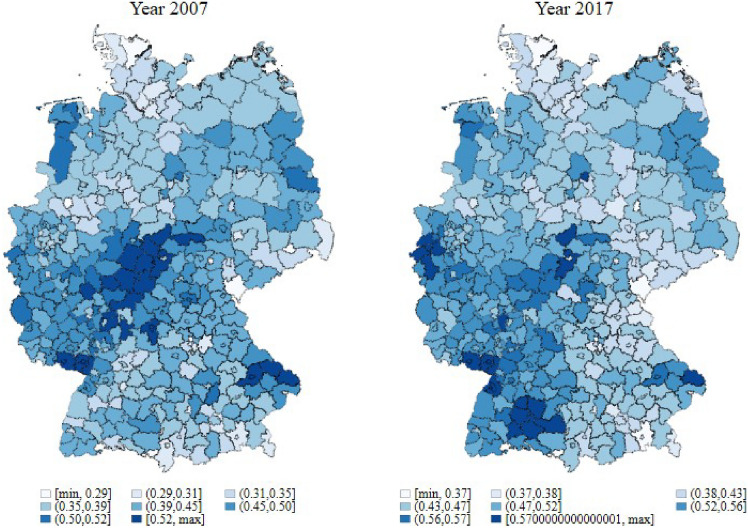
Share of elderly in informal care to all recipients over 65 across German counties in 2007 and 2017. *Sources*: RDC of the Federal Statistical Office and Statistical Offices of the Länder, Care Statistic, 2007 and 2017 (DOI: https://doi.org/10.21242/22411.2007.00.02.1.1.0–https://doi.org/10.21242/22411.2017.00.02.1.1.0), own calculations. *Note:*Regional variation of the share of people in informal care to all care recipients in Germany in 2007 (left) and 2017 (right) for different percentiles (p10, p25, p50, p75, p90). For data security reasons, we cannot show the maximum or minimum

## Data Availability

The Care Statistics are publicly available and can be accessed via the Statistical Office of the Länder under the contracted restrictions (e.g., on-site use and fees). DOI: 10.21242/22411.2007.00.02.1.1.0–10.21242/22411.2017.00.02.1.1.0.

## Appendix

### Nursing care quality index

The Medical Review Boards (MRB or MDK in German) of the German Statutory Health Insurance regularly inspect the quality of NHs (compare footnote 2 for more details on the bodies). Until 2019, the assessment was based on 77 criteria, formally divided into four broad categories: “Care and medical care”, “Dealing with residents with dementia,” “Care and everyday life,” and “Housing, nutrition services, housekeeping, and hygiene.” The MRB assessed NHs based on audits and surveys of the care recipients. Auditors randomly selected and interviewed up to nine care recipients of each provider and noted how many of the inspection criteria had been met. Then, the MRB translated this into school grades from 1 to 5 [44]. These grades were averaged across all criteria. This procedure had been largely criticized by professionals since average school grades can hide single indicators with insufficient quality. Hence, we follow Herr and Hottenrott [45] and Herr et al. [46] and translate the assessment outcomes into a stricter grade. First, we select seven criteria that reflect nursing quality. Second, for each criterion, we assign the value of 1 if it is fulfilled for *all* inspected residents and zero otherwise. Subsequently, we construct our quality index (*Q*) as an average over the selected criteria and obtain values between zero (none is fulfilled for all care recipients) and one (all are fulfilled for all care recipients).

4 $${Q}=\frac{1}{7}\sum_{i=1}^{7}{q}_{i} i = 1, \dots , 7$$

The nursing quality comprises the following seven survey questions (own translation into English):

1. Are the records for the treatment of chronic wounds or pressure ulcers (e.g., wound documentation) evaluated, and if necessary, is the doctor informed, and are the measures adjusted?
2. Is the nutritional status appropriate within the scope of the facility’s capabilities?
3. Is the liquid supply adequate within the scope of the facility’s capabilities?
4. Has a systematic pain assessment been conducted?
5. Are individual risks and resources assessed for residents with urinary incontinence or with urinary catheters?
6. Is the individual risk of falling assessed?
7. Do consents or approvals exist for freedom-restricting measures?

### Figures

See Figs. 4, 5, 6, 7, 8

### Tables

See Tables 5, 6, 7

**Table 5:** Description of explanatory variables and sources, aggregated at county level

	Description	Datasource
NHCutilization		
Elderly care recipients in NH	Share of people in need of care above 65 using NHC	Care statistic
Care needs		
Elderly care recipients in care level 1–3 across all care types	Share of people in need of care in care levels 2 and 3, compared to care level 1	Care statistic
Elderly care recipients aged 75–84 (85+), share	Share by age group, reference category: 65–74 aged care recipients	Care statistic
Predisposing		
Labor force participation [%]	Share of employed individuals in the county per 100 inhabitants of working age (15–65)	INKAR
Inhabitants, by age groups, [%]	Share of total population by age, $<34$ is the reference category	INKAR
Avg. life expectancy at 60 [years]	Average life expectancy for a 60-year-old person	INKAR
Enabling		
GDP per capita [1,000 EUR]	Gross Domestic Product (GDP) per inhabitant	INKAR
Use of social aid for the elderly [%]	Share of the population receiving basic elderly support	INKAR
Avg. pension [EUR]	Average monthly pension payment in euros	INKAR
Monthly household income per capita [EUR]	Average disposable household income in euros per inhabitant	INKAR
Rurality ** [%]	Share of a county’s inhabitants living in municipalities with less than $<150$ inhab. per km$^{2}$	INKAR
Communal debts per capita [EUR]	Communal debts per inhabitant	INKAR
LTC supply		
Hospital beds [per 10,000 inh.]	Hospital beds per 10,000 inhabitants	INKAR
Single room share in NH [%]	Average share of single rooms in NH	Care statistic
Occupancy rate	Share of residents per NH bed	Care statistic
Personnel to resident ratio	Share of full-time equivalent personnel to all residents	Care statistic
Home care facilities [#]	Number of home care providers (ambulatory care)	Care statistic
Nurse vacancy ratio	Share of job openings for geriatric care nurses to all budgeted positions	IAB
Average OOP by care level [EUR]***	Out-of-pocket contribution by care level (=NH price−LTC insurance payment as of Table 6)	Care statistic
Nursing care quality	Index of nursing care quality (see Equation 4 for further information)	Transparency reports

*Data sources*: *Care statistic* Care statistic, (DOI: 10.21242/22411.2007.00.02.1.1.0–10.21242/22411.2017.00.02.1.1.0); *INKAR* database of the Federal Office for Building and Regional Planning (BBR); *IAB* German Institute for Employment Research; *Transparency reports* Transparency report cards from the BKK comparison engine for stationary LTC: https://pflegefinder.bkk-dachverband.de/ [last accessed Sept. 2024]

**Table 6:** Long-term care insurance funds’ allowance

	2007	2009	2011	2013	2015	2017*
Informal care						
Care level I	205	215	225	235	244	316
Care level II	410	420	430	440	458	545
Care level III	665	675	685	700	728	728
Hardship case	–	–	–	–	–	901
Home health care						
Care level I	384	420	440	450	468	689
Care level II	921	980	1040	1100	1144	1298
Care level III	1432	1470	1510	1550	1612	1612
Hardship case	1918	1918	1918	1918	1995	1995
Nursing home care						
Care level I	1023	1023	1023	1023	1064	770
Care level II	1279	1279	1279	1279	1330	1262
Care level III	1432	1470	1510	1550	1612	1775
Hardship case	1688	1750	1825	1918	1995	2005

We present the maximum monthly allowance (euros) paid by the German public long–term care insurance for informal care, home health care, and nursing home care. Depending on their contributions, care recipients with private insurance are entitled to higher allowances. *Sources:* Bundesgesundheitsministerium; Pflegestärkungsgesetze I–III, Pflege-Neuausrichtungs-Gesetz. 2007-2011 (pp. 44-45): https://www.bundesgesundheitsministerium.de/fileadmin/Dateien/5_Publikationen/Pflege/Berichte/5.Pflegebericht.pdf, 2011–2015 https://www.bundesgesundheitsministerium.de/fileadmin/Dateien/5_Publikationen/Pflege/Berichte/6.Pflegebericht.pdf (pp. 116–117) and https://www.bundesgesundheitsministerium.de/ministerium/meldungen/2016/dezember-2016/neuregelungen-2017.html for 2017 [last accessed Nov. 2024]

**Table 7:** Spatial regression results based on OLS, model 5, 2007–2017, full results

	SLM	SEM	SAC
*Caregiving needs*			
Care recipients in care level 2, share	0.0153	0.0114	0.0154
Care recipients in care level 3, share	0.2468***	0.3443***	0.3476***
Share LTC recipients aged 75–84	– 0.4886***	– 0.4742***	– 0.4712***
Share LTC recipients aged 85+	0.7536***	0.7402***	0.7225***
*Predisposing*			
Female labor force participation, share	0.2801***	0.2707***	0.2773***
Male labor force participation, share	– 0.2143***	– 0.1975***	– 0.2091***
Share inhabitants $35-49$	0.1545	0.1678	0.1040
Share inhabitants aged $50-64$	– 0.3499***	– 0.3886***	– 0.4043***
Share inhabitants $65-74$	– 0.6831***	– 0.3948**	– 0.4215**
Share inhabitants $75-84$	1.4752***	1.2300***	1.2138***
Share inhabitants $\ge 85$	– 1.9391***	– 1.9298***	– 1.7317***
Avg. life expectancy at 60	– 0.0316***	– 0.0317***	– 0.0315***
*Enabling*			
Log (GDP per capita [1,000 EUR])	0.0031	0.0022	0.0030
Use of social aids for elderly	– 0.0017	0.0164	0.0218
Log (Avg. pension)	0.1355***	0.1612***	0.1507***
Share rurality**	– 0.0234***	– 0.0182***	– 0.0192***
Log (Available household income)	0.0220*	0.0205	0.0220*
Log (Communal debts [EUR])	– 0.0001	0.0001	– 0.0001
*Supply*			
Hospital beds [per 10,000 inh.]	0.0023***	0.0019***	0.0021***
Single room share in NH	– 0.0300***	– 0.0339***	– 0.0320***
Ratio ambulatory facilities per care recipient	– 0.0002***	– 0.0002***	– 0.0002***
Occupancy rate in NH	– 0.1576***	– 0.1507***	– 0.1510***
Vacancy ratio****	– 0.3091***	– 0.3332***	– 0.3334***
Nurses per resident	– 0.1187***	– 0.1249***	– 0.1195***
Log(Average OOP*** [EUR], Care level 1)	0.0967**	0.1277***	0.1237***
Log(Average OOP*** [EUR], Care level 2)	– 0.1306**	– 0.1056	– 0.1053*
	(0.056)	(0.064)	(0.064)
Log(Average OOP*** [EUR], Care level 3)	– 0.0030	– 0.0553	– 0.0523
Nursing care quality*****	0.0197**	0.0168**	0.0177**
Constant	2.3142***	2.1161***	2.1749***
Lambda ($\lambda$)	0.0060***		0.0065***
	(0.001)		(0.002)
Rho ($\rho$)		0.0823***	0.0770***
		(0.006)	(0.006)
MRB FE, Time FE	Yes	Yes	Yes
Observations	2,221	2,221	2,221

$$^{*}p<0.10$$, $$^{**}p<0.05$$, $$^{***}p<0.01$$. Standard errors suppressed for ease of presentation

Specifications: (1) Spatial Lag Model (SLM), Equation (2), (2) Spatial Error Model (SEM) Equation (3), (3) Spatial Autoregressive Combined Model (SAC), Equations (2) and (3).

Sources: Research Data Centre (RDC) of the Federal Statistical Office and Statistical Offices of the Länder, Care Statistics, survey years 2007–2017 (DOI: 10.21242/22411.2007.00.02.1.1.0–10.21242/22411.2017.00.02.1.1.0); INKAR database of the Federal Office for Building and Regional Planning (BBR). Own calculations

## Footnotes

1 In 2017, two levels were added, which we map to the former system the following way: the new care grades 1 and 2 correspond to level 1, care grade 3 to level 2, and care grades 4 and 5 to level 3.

2 The MRB is an independent non-profit organization providing socio-medical specialist advice to the German Statutory Health Care and LTC Insurance. An overview of federal state LTC regulations is available at http://www.biva.de/gesetze/laender-heimgesetze/, last accessed in July 2024.

3 Distances are calculated based on centroid coordinates provided by the German Office for Cartography and Geodesy (BKG), publicly available at http://www.geodatenzentrum.de/auftrag1/archiv/vektor/vg2500/, last accessed in July 2024.

4 The Moran-I coefficient is calculated as $I=\frac{N}{{\mathrm{\Sigma }}_{\mathrm{i}}{\mathrm{\Sigma }}_{\mathrm{j}}\mathrm{w}\mathrm{i}\mathrm{j}}\cdot \frac{{\mathrm{\Sigma }}_{\mathrm{i}}{\mathrm{\Sigma }}_{\mathrm{j}}\mathrm{w}\mathrm{i}\mathrm{j}\left({\mathrm{x}}_{\mathrm{i}}-\stackrel{-}{\mathrm{x}}\right)\left({\mathrm{x}}_{\mathrm{j}}-\stackrel{-}{\mathrm{x}}\right)}{{\mathrm{\Sigma }}_{\dot{\mathrm{i}}}{\left({\mathrm{x}}_{\mathrm{i}}-\stackrel{-}{\mathrm{x}}\right)}^{2}}$, where *N* is the number of spatial units, *w*_*ij*_ is an element of the spatial contiguity weight matrix, *X* is the vector of covariates, and *X*^¯^ is the mean of *X* [35].

5 Controlling for spatial dependencies only slightly changes the coefficient estimates, where all of them are similarly statistically (in-)significant, except that the share of elderly using social aid and the OOP for care level 2 turn into being weakly statistically significantly different from zero.

6 The share of elderly care recipients is not significantly correlated with usage compared to care level 1.
